# *clpC*-Mediated Translational Control Orchestrates Stress Tolerance and Biofilm Formation in Milk-Originated *Staphylococcus aureus* RMSA24

**DOI:** 10.3390/foods14244333

**Published:** 2025-12-16

**Authors:** Maofeng Zhang, Jie Hu, Ting Xue

**Affiliations:** 1 School of Life Science, Anhui Agricultural University, Hefei 230036, China; zmf19921101@163.com (M.Z.);; 2 Joint Research Center for Food Nutrition and Health of IHM, Anhui Agricultural University, Hefei 230036, China

**Keywords:** pathogenic bacteria, raw milk, *clpC*, biofilm, environmental stress

## Abstract

*Staphylococcus aureus* is an important pathogen that can cause widespread infections as well as severe outbreaks of food poisoning. Recent studies have drawn attention to foodborne pathogens such as *S. aureus* endowed with the ability to form biofilms and increase resistance to antimicrobial agents as well as environmental stress, posing challenges to food safety. The Clp (caseinolytic protease) protein complex plays a crucial role in energy-dependent protein hydrolysis processes. This mechanism is a common way to maintain intracellular homeostasis and regulation in both prokaryotic and eukaryotic cells, especially under stress conditions. In *S. aureus*, multiple genes encoding Clp ATPase homologues have been identified: *clpC*, *clpB*, *clpY*, *clpX*, and *clpL*. This study investigated the roles of *clpC* in stress tolerance and biofilm formation of foodborne *S. aureus* RMSA24 isolated from raw milk. Our results showed that the deletion of the *clpC* gene significantly reduced the bacterium’s tolerance to heat, desiccation, hydrogen peroxide, and high osmotic pressure compared to wild type (WT). Furthermore, the *clpC* knockout mutant also exhibited a marked decrease in biofilm formation using Crystal Violet Staining (CVS) and Scanning Electron Microscopy (SEM). Finally, compared to WT, there was a total of 102 DEGs (differentially expressed genes), with a significant downregulation of genes related to biofilm formation *(isaA* and *spa*) and heat-shock response (*clpP* and *danJ*). These findings suggest that *clpC* regulates environmental tolerance in *S. aureus* by modulating the expression of stress- and biofilm-related genes, positioning it as a potential biomarker and a novel target for controlling contamination in the food industry.

## 1. Introduction

*Staphylococcus aureus*, a Gram-positive facultative anaerobe, colonizes ~30% of humans and dairy cattle [1,2] and frequently contaminates milk products, producing heat-stable enterotoxins that survive pasteurization and rank it among the top five causes of global foodborne outbreaks [3,4]. Beyond intoxication, the emergence of methicillin-resistant *S. aureus* (MRSA) sequence type (ST) 398 in bulk-tank milk and the recent isolation of vancomycin-intermediate (VISA) and linezolid-resistant strains from dairy herds underline the public health and economic relevance of this pathogen [5,6]. *S. aureus* persists in foods through broad physiological tolerance and polysaccharide intercellular adhesin (PIA)/eDNA/amyloid biofilms that greatly enhance desiccation and heat and oxidative resistance [7,8,9].

Milk is a fundamental raw material for various dairy products, such as pasteurized milk, cheese, and milk powder [9]. Food processing such as heat treatment, freeze-drying, and vacuum concentration are used to kill the harmful bacteria and extend the shelf life of dairy products [10]. Additionally, hydrogen peroxide (H_2_O_2_) is an excellent disinfectant that can effectively inactivate foodborne pathogens. Despite these measures, food poisoning caused by *S. aureus* in dairy products remains a significant concern. Most pathogens are killed during food processing (high temperature, drying, and oxidation), but occasionally there may be residual bacteria [11]. Biofilm formation represents a critical survival strategy that enables bacteria to withstand environmental stresses through structured community development and extracellular matrix production. Biofilms can enhance the immune capacity of bacteria, protecting them from adverse environmental conditions and increasing their survival rate [12]. *Staphylococcus aureus* has a strong biofilm formation ability [13]. Therefore, there are many reports of food poisoning caused by *S. aureus* in dairy products.

Clp ATPase constitutes a family of proteins that are highly related and widely present. The Clp ATPase family is a member of the AAA+ superfamily. A defining characteristic of this family is the presence of a conserved region comprising approximately 220 amino acid residues, commonly known as the AAA domain. This domain harbors several highly conserved motifs, among which the Walker A and Walker B motifs are critically involved in ATP-binding and hydrolysis, respectively, serving as essential sequence elements for the enzymatic activity of ATPases [14]. Members of the Clp ATPase family are classified based on whether they possess one or two ATP-binding domains. The first type of Clp proteins have two ATP-binding sites, namely ATP-1 and ATP-2, and are relatively large (with a size range of approximately 70 to 110 kDa). The length variation in the intervening region connecting ATP-1 and ATP-2, as well as the appearance of specific characteristic sequences, form the basis for classifying them into ClpA, ClpB, ClpC, ClpD, ClpE, and ClpL, which are the Clp 1 ATPase family members. The smaller, second type of Clp proteins, such as ClpX and ClpY, contain only one ATP-binding site and have the greatest similarity to ATP-2 [13,14]. The Clp protein complex plays an indispensable role in the regulation of cellular protein quality and the survival of the cell in *S. aureus* [15,16]. Furthermore, research has shown that the CLP protease plays a crucial role in the pathogenicity of the virulence factors of *S. aureus* [17,18].

Given the central role of protein synthesis in stress adaptation, we hypothesized that *clpC* might coordinate the translational reprogramming required for *S. aureus* to withstand food-relevant stresses and to build protective biofilms. To test this, we constructed an in-frame *clpC* deletion in the raw milk isolate *S. aureus* RMSA24 and quantified its (i) survival under desiccation, heat, H_2_O_2_, and high-osmolarity challenges, (ii) biofilm biomass and architecture, and (iii) genome-wide transcriptional response.

## 2. Materials and Methods

### 2.1. Bacterial Strain

The *S. aureus* strain RMSA24 was isolated from raw milk samples procured from a dairy store in Hefei, Anhui, China, and stored at −80 °C in Trypticase Soy Broth (TSB) (Sangon Biotech, Shanghai, China) containing 25% glycerol. Routine cultivation was performed in TSB or TSB agar media at 37 °C. All the obtained strains and plasmids used in this study are listed in Table 1.

### 2.2. Construction of the clpC-Deficient Mutant and Growth Curve Analysis

We used shuttle plasmid pBT2 to inactivate *clpC* based on homologous recombination. Firstly, the upstream and downstream fragments flanking *clpC* were PCR-amplified from RMSA24 genomic DNA with the primers *clpC*-up-HindIII-F/*clpC*-up-R and *clpC*-down-F/*clpC*-down-BamHI-R, respectively. The erythromycin resistance (ermB) was amplified from plasmid pEC1 by the primer *clpC*-ermB-F/*clpC*-ermB-R. All of the PCR products were ligated using upstream and downstream fragments and ermB cassette fragments as a template with *clpC*-up-HindIII-F/*clpC*-down-BamHI-R by overlapping PCR. The products were digested with HindIII/BamHI and then ligated to the pBT2 plasmid using T4 ligase (Thermo Fisher Scientific). Firstly, the plasmid was transferred into *S. aureus* RN4220 for modification and then the modified plasmid was transferred into *S. aureus* RMSA24. After transforming the constructed shuttle plasmid pBT-*clpC* into a wild-type strain, the upstream and downstream homologous arms of *clpC* on the plasmid were double hybridized with the genome sequence of the wild-type strain according to the principle of homologous recombination. Then, the RMSA24Δ*clpC* strain was obtained through cultivation and antibiotic screening [12]. All the primers used in this study are shown in Table 2.

The WT RMSA24 and RMSA24Δ*clpC* strains were inoculated into TSB medium and cultured until the exponential phase. Then, the strains were diluted with OD_600_ to 0.03 followed by continued culturing. The absorbance value at OD_600_ at different times was determined by a UV spectrophotometer. The growth curve was plotted based on the absorbance at different times.

### 2.3. Desiccation Survival Assay

Overnight cultures of WT RMSA24 and RMSA24Δ*clpC* were diluted to OD_600_ = 0.03 in fresh TSB and grown to mid-exponential phase at 37 °C for 4 h. Both the cultures (50 μL of each) were put in the drying oven at 37 °C for 24 h and 48 h, respectively. The colony-forming units (CFUs) were measured on TSB agar medium before and after drying; survival rates were calculated as (CFU_post-dry/CFU_pre-dry) × 100%. All assays were performed in triplicate [12].

### 2.4. High-Temperature Survival Assay

Overnight cultures of WT RMSA24 and RMSA24Δ*clpC* were diluted to OD_600_ = 0.03 in fresh TSB and grown to mid-exponential phase at 37 °C for 4 h. Both the cultures were taken as 100 μL and put in the drying oven at 58 °C for 10 min and 30 min. Using the 10-fold gradient dilution method, CFU measurements were performed on bacteria before and after high-temperature treatment to obtain bacterial survival ability. All assays were performed in triplicate [12].

### 2.5. H_2_O_2_ Pressure Survival Assay

The WT RMSA24 and RMSA24Δ*clpC* strains were inoculated into TSB medium and cultured until the stationary phase. A total of 880 mM of H_2_O_2_ was added to the culture followed by continued culturing for 30 min and 60 min. Then, the number of viable bacteria with dilution coating was calculated. All assays were performed in triplicate [12].

### 2.6. High-Osmotic Pressure Survival Assay

Exponential-phase cultures of WT RMSA24 and RMSA24Δ*clpC* were inoculated into TSB with different concentration NaCl (0%, 5%, 10%, 15%, and 20%) with an initial OD_600_ = 0.05 and incubated at 37 °C with shaking (200 rpm). OD_600_ was recorded every 2 h for 12 h in a microplate reader; three independent wells were measured at each time point [12].

### 2.7. Biofilm Formation

Briefly, the exponential-phase cultures (OD_600_) were diluted into fresh TSB supplemented with 1% glucose, transferred to a 96-well plates, and incubated at 37 °C for 24 h. Adherent bacteria were stained with crystal violet and washed with PBS (Phosphate-Buffered Saline) (Sangon Biotech, Shanghai, China) (pH7.4). The stained cells were dissolved in 33% acetic acid, then the OD_492_ absorbance values were detected by microplate reader [12].

The WT RMSA24 and RMSA24Δ*clpC* were cultured on a sterile coverslip in a six-well plate (5 mL per well) at 37 °C for 24 h. After the incubation, the coverslip was removed and washed three times with PBS solution. The biofilm was fixed with 2.5% glutaraldehyde (Shanghai Sangjine Company, Shanghai, China) at 4 °C for 12 h and then dehydrated with ethanol solution (Sangon Biotech) for 20 min. Subsequently, the biofilm bacteria on the coverslip were frozen-dried for 12 h and fixed with precious metals. Finally, the biofilm bacteria were observed by SEM [12].

### 2.8. Transcriptome Analysis

Overnight cultures of WT RMSA24 and RMSA24Δ*clpC* were diluted to OD_600_ = 0.03 in fresh TSB and grown to mid-exponential phase (4 h, 37 °C, 200 rpm). Total RNA was extracted with TRIzol (TransGen Biotech, Beijing, China), rRNA was depleted, and strand-specific libraries were prepared and sequenced by Biozeron Biotechnology Co., Ltd. (Shanghai, China). The transcriptome analysis was tested [19].

RNA extraction: Total RNA was extracted from the tissue using TRIzol^®^ Reagent according to the manufacturer’s instructions (TransGen Biotech, Beijing, China and genomic DNA was removed using DNase I (TaKara, Beijing, China). Then RNA quality was determined using a 2100 Bioanalyser (Agilent, Santa Clara, CA, USA) and quantified using the ND-2000 (Termo Fisher Scientifc, Waltham, MA, USA). A high-quality RNA sample (OD260/280 = 1.8~2.2, OD260/230 ≥ 2.0, RIN ≥ 6.5, 28S:18S ≥ 1.0, >10 μg) was used to construct a sequencing library.

Library preparation and Illumina Hiseq sequencing: RNA-seq strand-specific libraries were prepared following the TruSeq RNA sample preparation kit from Illumina (San Diego, CA, USA), using 5 μg of total RNA. Briefly, rRNA removal was performed using the RiboZero rRNA removal kit (Epicenter) and fragmented using fragmentation buffer. cDNA synthesis, end repair, A-base addition, and ligation of the Illumina-indexed adaptors were performed according to Illumina’s protocol. Libraries were then size-selected for cDNA target fragments of 200–300 bp on 2% Low Range Ultra Agarose followed by PCR amplification using Phusion DNA polymerase (NEB) for 15 PCR cycles. After being quantified by TBS380, paired-end libraries were sequenced by Illumina NovaSeq 6000 sequencing (150 bp*2, Shanghai BIOZERON Co., Ltd., Shanghai, China).

Differential expression analysis and functional enrichment: To identify DEGs (differentially expression genes) between the two different samples, the expression level for each transcript was calculated using the fragments per kilobase of read per million mapped reads (RPKM) method. edgeR (https://bioconductor.org/packages/release/bioc/html/edgeR.html, accessed on 14 August 2021) was used for differential expression analysis. The DEGs between two samples were selected using the following criteria: (i) the logarithmic of fold change was greater than 2 and (ii) the false discovery rate (FDR) was less than 0.05. To understand the functions of the differentially expressed genes, GO (Gene Ontology) functional enrichment and KEGG (Kyoto Encyclopedia of Genes and Genomes) pathway analysis were carried out by Goatools (https://github.com/tanghaibao/Goatools, accessed on 14 August 2021) and KOBAS (http://kobas.cbi.pku.edu.cn/home.do, accessed on 14 August 2021), respectively. DEGs were significantly enriched in GO terms and metabolic pathways when their Bonferroni-corrected *p*-value was less than 0.05.

## 3. Results

### 3.1. Construction of clpC Knockout Strain

**Figure 1:**
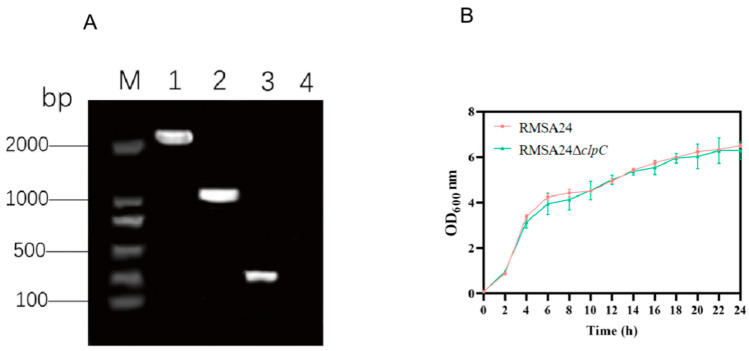
Identification of the *clpC* mutant strain RMSA24Δ*clpC* (**A**): M: 2000 bp DNA marker; lane 1: WT; lane 2: RMSA24Δ*clpC*; lane 3: WT; lane 4: RMSA24Δ*clpC*; (**B**): Growth curves of strains RNSA24 and RMSA24Δ*clpC*.

Genetic verification of *clpC* knockout in the RMSA24 PCR-based mutant strain: Using primers flanking the *clpC* locus, wild-type RMSA24 yielded a PCR product exceeding 2000 bp (Figure 1A, lane 1), and this product was the gene *clpC.* The mutant strain produced a ~1000 bp amplicon corresponding to the ermB resistance cassette insertion (Figure 1A, lane 2). Internal validation using check_*clpC*_in-F/check_c*lpC*_in-R primers specific to the *clpC* coding sequence amplified the expected ~250 bp product from wild-type RMSA24 (Figure 1A, lane 3) but yielded no product from the mutant strain RMSA24Δ*clpC* (Figure 1A, lane 4); this product was a gene fragment in *clpC*, confirming complete deletion of the target gene. DNA sequencing of the PCR products further verified the precise replacement of *clpC* with the ermB resistance marker [12].

To assess the impact of *clpC* deletion on bacterial fitness, growth kinetics of the RMSA24 and RMSA24Δ*clpC* strains were monitored spectrophotometrically at OD_600_. As shown in Figure 1B, the RMSA24Δ*clpC* mutant during the whole growth cycle was basically the same as that of the wild strain RMSA24, with both strains displaying similar lag phase duration, exponential growth rates, and maximum cell densities. These results indicate that *clpC* is dispensable for normal growth under the tested laboratory conditions.

### 3.2. Effect of clpC Knockout on Desiccation Tolerance in RMSA24

**Figure 2:**
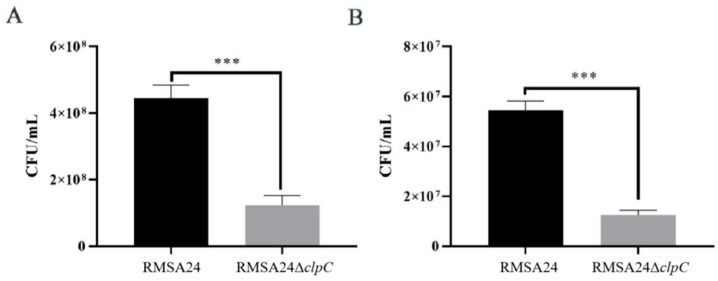
Viable counts of RMSA24 and RMSA24Δ*clpC* strains before and after drying treatment ((**A**): 24 h; (**B**): 48 h). ***, *p* < 0.001.

Dairy processing typically involves drying treatment to facilitate long-term storage and transportation. For this, we conducted controlled drying experiments comparing the viability of wild-type RMSA24 and the RMSA24Δ*clpC* mutant strain. Following dehydration, viable cell counts were determined by serial dilution plating at multiple time points. As demonstrated in Figure 2, significant differences in survival capacity were observed between the two strains. After 24 h of desiccation (Figure 2A), the *clpC* mutant exhibited a marked reduction in viability compared to the parental strain. This differential survival phenotype became even more pronounced after 48 h of drying (Figure 2B), indicating that the absence of *clpC* compromises the bacterium’s ability to withstand prolonged dehydration stress. Quantitative analysis revealed that the CFU of RMSA24Δ*clpC* was reduced 3.5-fold and 4.3-fold compared to wild-type RMSA24 after 24 and 48 h of desiccation, respectively.

### 3.3. Effect of clpC Knockout on Thermotolerance in RMSA24

**Figure 3:**
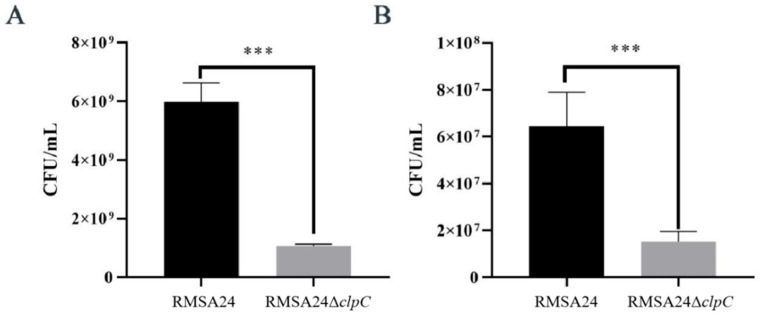
Viable counts of RMSA24 and RMSA24Δ*clpC* strains before and after high-temperature treatment ((**A**): 10 min; (**B**): 30 min). ***, *p* < 0.001.

To investigate the role of *clpC* in bacterial thermotolerance, we assessed the survival capacity of wild-type RMSA24 and RMSA24Δ*clpC* under high-temperature stress conditions. As demonstrated in Figure 3, RMSA24Δ*clpC* exhibited significantly compromised thermotolerance compared to the parental strain. Following 10 min of heat exposure, RMSA24Δ*clpC* showed a 5.6-fold reduction in viable cell counts relative to wild-type RMSA24 (Figure 3A). This thermosensitive phenotype was sustained with prolonged heat stress, as the mutant strain displayed a 4.2-fold decrease in CFU after 30 min of high-temperature treatment (Figure 3B).

### 3.4. Effect of clpC Knockout on Oxidative Stress Resistance in RMSA24

**Figure 4:**
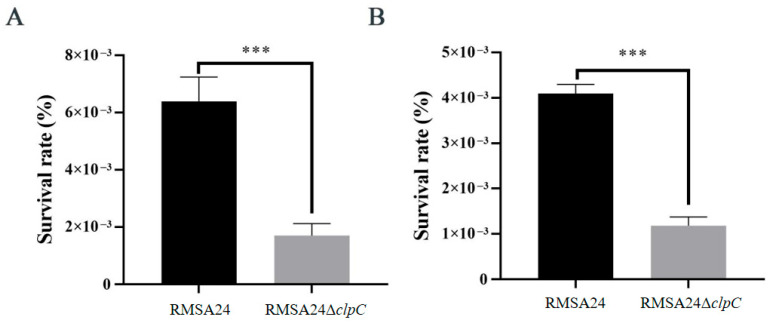
Survival rate of strains RMSA24 and RMSA24Δ*clpC* after H_2_O_2_ treatment ((**A**): 30 min; (**B**): 60 min). ***, *p* < 0.001.

To investigate the role of *clpC* in bacterial oxidative stress resistance, we assessed the survival capacity of RMSA24 and RMSA24Δ*clpC* under H_2_O_2_ exposure. Both strains were challenged with 880 mM of H_2_O_2_ under standardized conditions, and viability was monitored over time. Following 30 min of H_2_O_2_ exposure, RMSA24Δ*clpC* showed markedly reduced survival rates compared to wild-type RMSA24 (Figure 4A). This sensitivity to oxidative stress was sustained with prolonged exposure, as the mutant strain continued to display significantly lower viability after 60 min of H_2_O_2_ treatment (Figure 4B).

### 3.5. Effect of clpC Knockout on Hyperosmotic Stress Tolerance in RMSA24

**Figure 5:**
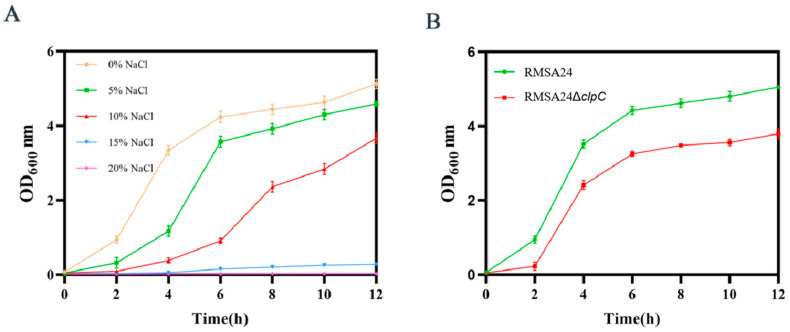
Effect of different concentrations of NaCl on the growth of strains RMSA24 and RMSA24Δ*clpC* ((**A**): different concentrations of salt added; (**B**): with 5% NaCl added).

We first established the salinity tolerance threshold of the parental RMSA24 strain by monitoring growth across a NaCl concentration gradient (0–20% *w*/*v*) in TSB medium. As shown in Figure 5A, NaCl exposure exerted a concentration-dependent inhibitory effect on RMSA24 growth, with complete growth arrest observed at 20% NaCl. This concentration-dependent inhibition aligns with established mechanisms where hyperosmotic conditions impair bacterial growth through plasmolysis, protein denaturation, and disruption of cellular homeostasis. Based on these baseline data, we selected 5% NaCl (approximately 0.85 M) as a sub-lethal stress condition to evaluate the contribution of *clpC* to osmotic stress tolerance. Under these standardized hyperosmotic conditions, the RMSA24Δ*clpC* mutant exhibited significantly impaired growth compared to the wild-type strain (Figure 5B). The mutant strain displayed extended lag phase duration and reduced exponential growth rate, indicating that *clpC* deletion compromises the bacterium’s capacity to adapt to osmotic stress.

### 3.6. Effect of clpC Knockout on Biofilm Formation in RMSA24

**Figure 6:**
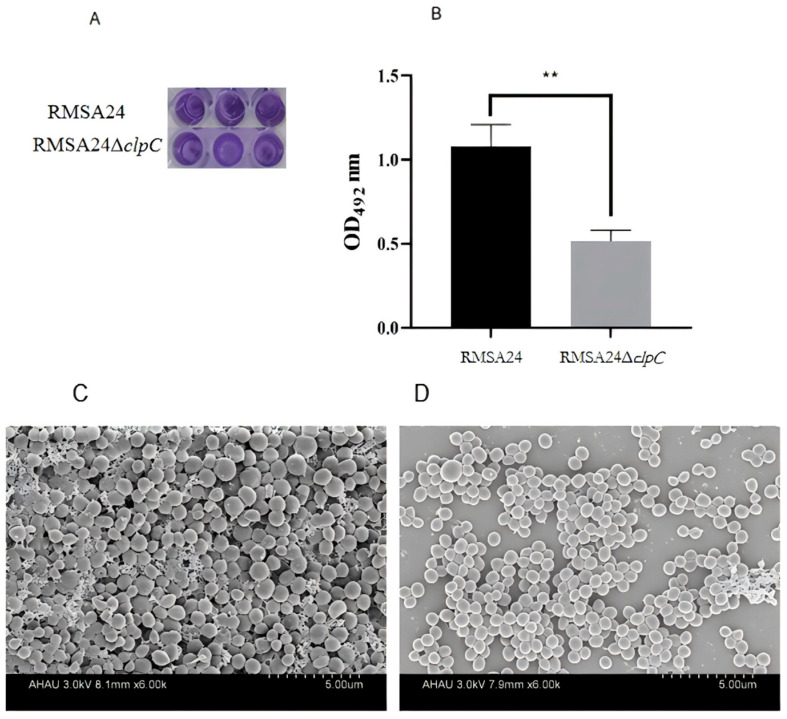
Detection of biofilm formation in strains RMSA24 and RMSA24Δ*clpC* ((**A**): a photograph of biofilms in the 96-well plates after staining with crystal violet; (**B**): CVS measured by optical density at 492 nm; (**C**): SEM of RMSA24; (**D**): SEM of RMSA24Δ*clpC*). **, *p* < 0.01.

We conducted a comprehensive analysis comparing biofilm formation capacity between wild-type RMSA24 and the RMSA24Δ*clpC* mutant strain using the standardized crystal violet assay. As demonstrated in Figure 6A, visual inspection revealed marked differences in biofilm biomass between the two strains. Spectrophotometric quantification at 492 nm confirmed that the wild-type RMSA24 produced significantly more biofilm biomass than the *clpC* knockout strain (Figure 6B). To further characterize the structural differences in biofilm architecture, SEM imaging revealed that wild-type RMSA24 formed dense, three-dimensional biofilm structures with extensive extracellular matrix production (Figure 6C). In contrast, the RMSA24Δ*clpC* mutant exhibited sparse, poorly developed biofilms with minimal extracellular matrix deposition (Figure 6D). This structural deficiency indicates that *clpC* deletion not only reduces total biofilm biomass but also compromises the development of mature biofilm architecture.

### 3.7. Transcriptomic Analysis of clpC Knockout in RMSA24

**Figure 7:**
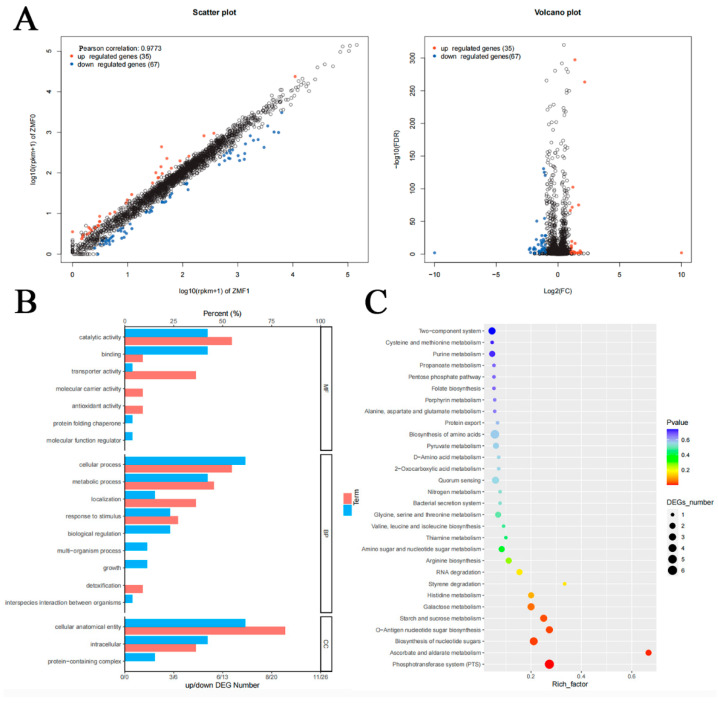
Differentially expressed genes in transcriptome analysis of RMSA24 and RMSA24Δ*clpC* ((**A**): visualization scatter plot and volcano plot display; (**B**): GO secondary annotation diagram; (**C**): KEGG scatter map).

To elucidate the molecular mechanisms underlying the phenotypes observed in the RMSA24Δ*clpC* mutant, we performed comprehensive transcriptomic analysis comparing gene expression profiles between RMSA24 and the *clpC* knockout strain. RNA sequencing revealed significant alterations in global gene expression patterns, with a total of 102 differentially expressed genes (DEGs) identified using stringent criteria (adjusted *p*-value ≤ 0.05 and fold change ≥ 1.5). Among these, 35 genes were significantly upregulated while 67 genes were downregulated in the *clpC* mutant (Figure 7A). Notably, several key stress response- and biofilm-associated genes exhibited differential expression patterns. The *clpC* mutation resulted in the downregulation of *isaA* (immunodominant surface antigen A) and *spa* (staphylococcal protein A), both of which are implicated in biofilm formation and bacterial adhesion. Additionally, *clpP* (Casein lytic proteinase P) and the molecular chaperone gene *dnaJ*, critical for environmental stress adaptation and heat-shock response, were significantly downregulated in the mutant strain. These transcriptomic changes align with our phenotypic observations of compromised biofilm formation and reduced stress tolerance in the *clpC* knockout strain.

To systematically characterize the functional implications of these transcriptional changes, we performed Gene Ontology (GO) enrichment analysis, which revealed 19 significantly enriched GO terms (Figure 7B). The most prominent category was “cellular anatomical entity,” consistent with the established role of *clpC* in maintaining cellular structural integrity and metabolic regulation. Kyoto Encyclopedia of Genes and Genomes (KEGG) pathway analysis identified 30 significantly enriched pathways, with metabolic pathways representing the most significantly affected functional category (Figure 7C).

## 4. Discussion

This study provides the first comprehensive characterization of clpC in *S. aureus* RMSA24 isolated from raw milk, revealing its critical role as a global regulator of stress tolerance and biofilm formation in this important foodborne pathogen. Our findings demonstrate that clpC functions as a central hub coordinating bacterial responses to multiple environmental stresses encountered during food production and preservation, including thermal stress, desiccation, oxidative challenge, and hyperosmotic conditions. In industrial production, bacteria are subject to various environmental stress factors, such as high temperature and high pressure [19]. The Clp protein complex is crucial for the reactivation and folding of damaged proteins under stress conditions [20]. The importance of the heat-shock proteins ClpX and ClpP of *S. aureus* has been confirmed in terms of stress resistance and pathogenicity [21,22]. Our data indicate that the mutant can enhance the sensitivity to heat shock. Additionally, the expression level of clpP in the transcriptome data decreases along with the mutation. From this, it can be inferred that clpC affects the emergency response under high-temperature conditions by influencing the expression of clpP.

It was reported that *clpC* cannot affect the resistance of *Staphylococcus aureus* to hydrogen peroxide [18]. But these experiments were conducted using bacteria that were in the exponential phase. However, when using the stationary phase strain, *clpC* can affect the hydrogen peroxide stress tolerance. Therefore, the growth stage seems to be important for the role of *clpC* on oxidative stress resistance. *clpP* plays a crucial role by degrading oxidized proteins to maintain the reduced environment in the cell [22]. Therefore, c*lpC* may affect the emergency response under oxidative stress resistance by influencing the expression of *clpP* based on the transcriptome results.

The substantial reduction in biofilm biomass and altered architectural development observed in the RMSA24Δ*clpC* mutant indicates that *clpC*-mediated translation is essential for the transition from planktonic to sessile lifestyle [23]. This phenotype was corroborated by transcriptomic data showing the downregulation of *isaA* and *spa*. The dynamic process of biofilm formation, involving attachment, maturation, and dispersion phases [24,25,26,27], appears to be disrupted at multiple stages in the absence of functional *clpC*. This finding is particularly relevant given the established correlation between biofilm formation and stress tolerance in *S. aureus* [28,29]. The integration of phenotypic data with transcriptomic analysis reveals that *clpC* functions as a global regulator coordinating multiple adaptive responses. The identification of 102 DEGs, with significant enrichment in metabolic pathways and cellular structural components, suggests that *clpC*-mediated translational control extends beyond stress response to encompass fundamental cellular processes. The downregulation of *isaA* and *spa* in RMSA24Δ*clpC* provides mechanistic insight into the biofilm defect, as these genes encode surface proteins essential for bacterial adhesion and biofilm structural integrity [30]. Similarly, the reduced expression of *clpP* and *dnaJ* explains the compromised thermotolerance, as *dnaJ* functions as a co-chaperone with *dnaK*, and *clpP* can degrade damaged or erroneous proteins in the heat-shock response system [22]. These findings suggest that *clpC* may facilitate the selective translation of stress-responsive mRNAs and can promote the expression of various proteins under stress conditions like IsaA, Spa, ClpP, and DnaJ. Therefore, this change affects the ability of stress resistance. This finding is consistent with previous studies demonstrating that *clpC* domains I, II, and III are highly conserved and essential for stress adaptation [31].

The ability of *S. aureus* to survive pasteurization and persist in dairy products represents a significant public health risk, as evidenced by numerous dairy-associated foodborne illness outbreaks [32,33,34,35]. Our findings indicate that *clpC* is essential for bacterial survival under food-processing conditions, which helps to identify this gene as a potential target for novel intervention strategies. The multi-stress resistance conferred by *clpC* suggests that targeting this protein could enhance the efficacy of existing food preservation methods. For instance, combination treatments that inhibit *clpC* function while applying thermal or osmotic stress could achieve synergistic bacterial inactivation. Furthermore, the identification of *clpC*-regulated genes such as *isaA*, *spa*, *clpP*, and *dnaJ* provides potential biomarkers for predicting bacterial survival capacity in food matrices.

## 5. Conclusions

This study establishes *clpC* as a previously unrecognized determinant of environmental robustness in *S. aureus* RMSA24 isolated from raw milk. Our findings showed that the deletion of *clpC* in the raw milk isolate RMSA24 decreased survival under heat, oxidative, osmotic, and desiccation challenges and reduced biofilm biomass and mean thickness. Transcriptome analysis revealed that the mutant downregulated the heat-shock chaperone gene *clpP* and *dnaJ* and the biofilm matrix genes *spa* and *isaA*, implicating *clpC* in a regulatory network that couples protein homeostasis to biofilm architecture. Our data indicate that *clpC* governs both stress-protective translation and the expression of adhesion/exopolysaccharide genes, positioning it as a dual-function node for intervention. Targeting *clpC* via small-molecule inhibitors or anti-*clpC* peptide–PNA antisense oligomers could weaken biofilm persistence and potentiate lethal sensitization, providing a next-generation strategy to curtail *S. aureus* contamination throughout the dairy continuum.

## Figures and Tables

**Table 1:** List of strains and plasmids used in this study.

Strains	Details	Reference or Source
*S. aureus*		
RMSA24	WT	Milk
RN4200	8325-4, restriction-negative strain	NARSA
*E. coli*		
DH5α	Clone host strain, supE44 ΔlacU169(φ80 lacZΔM15) hsdR17 recA1 endA1 gyrA96 thi-1relA1	Invitrogen (Waltham, MA, USA)
Plasmids		
pEC1	pBluescript derivative. Source of ermB gene, Amp^r^	Research group
pBT2	shuttle vector, temperature sensitive, Amp^r^, Cm^r^	Research group
pBT-*clpC*	pBT2 derivative, for clpC mutagenesis; Amp^r^, Cm^r^, Em^r^	This study

**Table 2:** Primers used in this study.

Primer Name	Oligonucleotide (5′–3′)
*clpC*-up HindIII-F	GCGAAGCTTTAACGCTTAATTGCTCA
*clpC*-up-R	GAAATTCCAGTCATGCAAGTGGTCA
*clpC*-ErmB-F	CATAAGCGAAATAGATTTAAAATTTCGC
*clpC*-ErmB-R	TGAAGTGTGGATACCATGCAAGTGGTCA
*clpC*-down-F	AGGATTCGGATTCAATGGCTCT
*clpC*-down *Bam*HI-R	GCGGGATCCACCGCTATACTCTTCGC
check-pBT2-F	TCACCGACAAACAACAGA
check-pBT2-R	CCAAGCCTATGCCTACA
check-*clpC*-in-F	GAACGGAAGTTTGAAGCC
check-*clpC*-in-R	TAAACCGATACGCTCACC
check-*clpC*-out-F	GTAGGTGCAGTTCGATTTCAGG
check-*clpC*-out-R	AAAAAGAAGCTGGTTCAGCTCT

Underline: the restriction endonuclease recognition sites.

## Data Availability

The original contributions presented in this study are included in the article. Further inquiries can be directed to the corresponding author.
